# How Context Influences Our Perception of Emotional Faces: A Behavioral Study on the Kuleshov Effect

**DOI:** 10.3389/fpsyg.2017.01684

**Published:** 2017-10-04

**Authors:** Marta Calbi, Katrin Heimann, Daniel Barratt, Francesca Siri, Maria A. Umiltà, Vittorio Gallese

**Affiliations:** ^1^Department of Medicine and Surgery, Unit of Neuroscience, University of Parma, Parma, Italy; ^2^Interacting Minds Centre, Aarhus University, Aarhus, Denmark; ^3^Department of Management, Society and Communication, Copenhagen Business School, Copenhagen, Denmark; ^4^Department of Food and Drug Sciences, University of Parma, Parma, Italy; ^5^Institute of Philosophy, School of Advanced Study, University of London, London, United Kingdom

**Keywords:** facial expressions, emotion, contexts, film editing, Kuleshov effect

## Abstract

Facial expressions are of major importance in understanding the mental and emotional states of others. So far, most studies on the perception and comprehension of emotions have used isolated facial expressions as stimuli; for example, photographs of actors displaying facial expressions corresponding to one of the so called ‘basic emotions.’ However, our real experience during social interactions is different: facial expressions of emotion are mostly perceived in a wider context, constituted by body language, the surrounding environment, and our beliefs and expectations. Already in the early twentieth century, the Russian filmmaker Lev Kuleshov argued that such context, established by intermediate shots of strong emotional content, could significantly change our interpretation of facial expressions in film. Prior experiments have shown behavioral effects pointing in this direction, but have only used static images as stimuli. Our study used a more ecological design with participants watching film sequences of neutral faces, crosscut with scenes of strong emotional content (evoking happiness or fear, plus neutral stimuli as a baseline condition). The task was to rate the emotion displayed by a target person’s face in terms of valence, arousal, and category. Results clearly demonstrated the presence of a significant effect in terms of both valence and arousal in the fear condition only. Moreover, participants tended to categorize the target person’s neutral facial expression choosing the emotion category congruent with the preceding context. Our results highlight the context-sensitivity of emotions and the importance of studying them under ecologically valid conditions.

## Introduction

Albeit there are many theories related to emotions and their comprehension, the present study focuses on the idea that facial expressions are of major importance in understanding the mental and emotional states of others (e.g., Tomkins, 1962–1963; Ekman, 1992, 1993; Izard, 1994; Russell, 1997). In this respect, two main approaches have been developed so far: the *categorical* and the *dimensional* approach (Aviezer et al., 2008). The categorical approach, in accordance with the facial dominance perspective (Tomkins, 1962–1963; Carroll and Russell, 1996), holds that basic facial expressions index specific emotions, reducible into clearly different categories (basic or primary emotions vs. complex or secondary emotions) (e.g., Ekman and Friesen, 1971; Ekman, 1992, 1993).

Conversely, the dimensional approach holds that emotions are defined by the different neurophysiological mechanisms of valence and arousal, the first mechanism referring to the degree of pleasantness/unpleasantness of an emotion, the second referring to the intensity of an emotion (which can vary from calm to excited). Thus, a certain emotion would be the result of a distinct combination of values of these two dimensions (e.g., Aviezer et al., 2008; Hajcak et al., 2011). In this vein, facial expressions convey information related to both the degree of pleasantness/unpleasantness (valence) and of physiological activation (arousal) (e.g., Russell, 1980, 1997).

As stressed by Aviezer et al. (2008), both approaches share the idea that *“affective information… is read out from the face by a process that is relatively immune to context”* (Aviezer et al., 2008, p. 724). This is the reason why most studies on the perception and comprehension of emotions so far have used isolated emotional facial expressions as stimuli. However, there is evidence that our real experience during social interactions in fact is not as independent from other influences (e.g., de Gelder et al., 2006). Already 20 years ago, for instance, Carroll and Russell (1996, p. 207) demonstrated that the same facial expression could convey different meanings depending on the context in which it was located: the perceiver can infer the emotion expressed by a facial expression referring to pleasantness, arousal and “quasi-physical” information, *all of which are interpreted in light of available information about the expresser’s situation.*

In the same vein, Wieser and Brosch (2012) highlighted how faces and facial expressions are always perceived in a wider context involving not only within-face features (e.g., eye gaze; Boll et al., 2011), but also within-sender features (e.g., body postures; Meeren et al., 2005; Aviezer et al., 2008), external features (e.g., emotional labels, verbal descriptions or visual scenes; Kim et al., 2004; Mobbs et al., 2006; Barrett et al., 2007; Schwarz et al., 2013), and within-perceiver features (e.g., personality traits; Calder et al., 2011). This is also in line with the *behavioral ecology view* of facial expressions (e.g., Fridlund, 1994), which stresses the relevance of situational context and communication (Barratt et al., 2016).

Already in the early twentieth century, the Soviet filmmaker Kuleshov (1899–1970) argued that such situational context could significantly change our interpretation of facial expressions. He designed an experiment in which he edited two close-ups of the Russian actor Ivan Mozzhukhin’s neutral face with three different emotional contexts: happy (a little girl playing with a doll), sad (a dead woman in a coffin), and hungry (a bowl of soup) (e.g., Pudovkin, 1970; Barratt et al., 2016). The viewers of the three film sequences reportedly perceived the actor’s neutral face as expressing an emotion congruent with the preceding context (Kuleshov, 1974; Barratt et al., 2016). The story has been passed on as a demonstration about contextual priming in movies, also known as the *Kuleshov effect* (Carroll, 1993).

Barratt et al. (2016) recently described in detail the characteristics of a Kuleshov-type sequence, arguing that it can be understood as a crossover between Soviet *montage editing* and classical *continuity editing*. In terms of the latter, a Kuleshov-type sequence can be regarded more precisely as an instance of *point-of-view* (POV) editing. A typical POV structure shows a first shot of a character looking off-screen in the direction of an object/event (glance-shot), followed by a second shot of the object/event in question (object-shot) (Branigan, 1984; Carroll, 1993; Persson, 2003; Barratt et al., 2016). When the object is presented from the perspective of the character, we have a “true POV” (Brewster, 1982; Persson, 2003; Barratt et al., 2016). The glance-shot can be either shown before or after the object, in what has been, respectively, called “prospective” and “retrospective” POV structure (Branigan, 1984; Carroll, 1993; Barratt et al., 2016).

To our knowledge, there have been only three previous attempts at replicating the original Kuleshov experiment. Prince and Hensley (1992) showed an actor’s neutral face, a static emotional image and the actor’s neutral face again, and then asked participants to evaluate the actor’s emotional performance, selecting from a list of emotions on a check-sheet: happiness, sadness, anger, fear, surprise, disgust, hunger, “no emotion,” and “other” (see the categorical approach; e.g., Ekman and Friesen, 1971). Results did not demonstrate a Kuleshov effect as most of participants chose the “no emotion” option, and those who reported perceiving emotions, chose an option unexpected with respect to the particular context.

More recently, Mobbs et al. (2006) revised the Kuleshov effect paradigm to investigate, by means of functional magnetic resonance imaging (fMRI), the neural correlates of contextual modulations on facial expression and mental-state attributions. Participants were asked to rate the emotional expression and mental state of a still image of a face, crosscut with an emotional image, using a two-dimensional rating scale (see the dimensional approach; e.g., Russell, 1980). Behavioral and fMRI results substantiated the Kuleshov effect with higher ratings of valence and arousal for faces paired with positive and negative contexts than for those paired with neutral contexts, and enhanced BOLD responses in several brain regions including the amygdala.

However, as stressed by Barratt et al. (2016), both studies showed limitations regarding the experimental design, that make comparisons difficult: while the negative results of Prince and Hensley (1992) could be attributed to problems of statistical power (single-trial experiment), the second study diverged from the traditional Kuleshov paradigm in various details.

Bearing in mind these limitations, Barratt et al. (2016) recently replicated the Kuleshov experiment with an improved experimental design attempting to respect as many rules as possible in order to increase the possibility that participants would infer that the glance shot and the object shot were spatially related (Persson, 2003; Barratt et al., 2016, p. 7). Thirty-six participants were presented with 24 film sequences of neutral faces (rendered dynamic with the zoom-in effect) paired with contexts belonging to six different emotional conditions (happiness, sadness, hunger, fear, desire, and “null condition”). As the contexts could be either static or dynamic objects, the authors used either a photograph with a slow zoom-in effect or a video clip. In order to combine the categorical and dimensional approach to emotion, participants were asked to rate both the valence and arousal of the target person’s emotion, and to explicitly categorize the type of emotion by choosing among different options. During the experiment, eye movements were recorded. Results showed significant behavioral effects pointing in the expected direction (from both a categorical and dimensional point of view). Specifically, neutral faces paired with sad contexts were rated as the most negative and least aroused, while neutral faces paired with desire contexts were perceived as the most positive and the most aroused (Barratt et al., 2016; pp. 15–16).

With the present study, we aimed at investigating and exploring further Barratt et al.’s (2016) results with some variations with respect to the original paradigm making up for an even more ecological design (for details, please see sections “Materials and Methods and Discussion”). Furthermore, we aimed at verifying the persistence of the effect despite these variations in order to employ the same experimental paradigm in a future electroencephalographic study to explore the contextual modulations on emotion processing at both the physiological and cortical levels. Participants were shown 18 film sequences of neutral faces crosscut with scenes evoking two different emotions (happiness, and fear, plus neutral stimuli as a baseline condition). Hence, from a dimensional point of view (e.g., Russell, 1980), we chose emotions characterized by distinct combination of values of these two dimensions: happiness (positive valence and medium arousal), fear (negative valence and high arousal) and neutral (neutral valence and low arousal) (see also Lang and Bradley, 2007). We employed only two emotional contexts (happy and fearful) in order to keep the design as simple as possible, and to highlight the differences between opposite emotional conditions in terms of valence. In particular, we adopted fear as a negative emotion because, from an evolutionary point of view, it is capable of directing our attention to potentially dangerous stimuli activating one of the two major motivation circuits (defensive vs. appetitive motivational systems; e.g., Bradley et al., 2001a,b; Lang and Bradley, 2010). Since we focused on both a dimensional and categorical approach to emotion (e.g., Ekman and Friesen, 1971; e.g., Russell, 1980) in order to have as much information as possible about participants’ experience (see also Barratt et al., 2016), we adopted happiness as a positive emotion. In comparison to desire (which is capable of activating the appetitive motivational system; Bradley et al., 2001a,b; Sabatinelli et al., 2001), happiness is more clearly reducible to one of the *basic* emotional categories (e.g., Ekman and Friesen, 1971). Thus, the task was to rate the emotion displayed by a target person’s face in terms of valence, arousal, and category. As contextual stimuli, we employed dynamic scenes in order to study the context-sensitivity of emotions under more ecologically valid conditions. We expected to find a significant difference between the ratings of valence, arousal, and category attributed to neutral faces paired with emotional contexts (both fearful and happy) and those attributed to neutral faces in neutral contexts. More specifically, we expected neutral faces in fearful contexts to be rated with more negative valence and higher arousal scores than neutral faces in neutral contexts, and neutral faces in happy contexts to be rated with more positive valence and higher arousal scores than neutral faces in neutral contexts.

## Materials and Methods

### Participants

Twenty-eight adult volunteers of Italian nationality took part in the study (14 female); mean age 28.1 years (standard deviation, *SD* = 4.7); age range: 22–40 years. All participants had normal or corrected-to-normal visual acuity. All participants provided a written informed consent to participate in the study, which had been approved by the Institutional Review Board of the University of Parma and has been conducted according to the principles expressed in the Declaration of Helsinki.

### Stimuli and Procedure

#### Stimuli

The stimuli consisted of film sequences created by editing together three different shots: the close-up of a target person’s neutral face (glance shot), followed by a view of the scene or event that the target person was looking at (object shot), followed by another close-up of the target person’s neutral face (glance shot) (Barratt et al., 2016).

##### Faces (glance shots)

To create the film sequences, we used the 24 neutral faces (12 female) selected and digitally manipulated by Barratt et al. (2016) from the Karolinska Directed Emotional Faces picture set (KDEF; Lundqvist et al., 1998). In contrast to the original study of Barratt et al. (2016), we selected a shorter shot (3-s long instead of 6-s) but kept the slow “zoom-in” effect. We then divided each shot in the middle, resulting in two 1.5-s shots. In this way, as recommended by Barratt et al. (2016), we guaranteed both the dynamic character of all shots and the spatiotemporal continuity between the opening and the closing glance shot. All of the faces were gray-scaled and presented in three-quarter profile in order to avoid a direct gaze into the camera and to facilitate the illusion that the person was looking at an object in an off-screen space (Barratt et al., 2016). Moreover, to control for potential confounding effects due to gaze direction and face orientation, we mirrored each face [half of the faces looked to the right (*N* = 24) and the other half looked to the left (*N* = 24)]. All of the faces had a resolution of 640 pixels × 480 pixels.

##### Emotional contexts (object shots)

**FIGURE 1:**
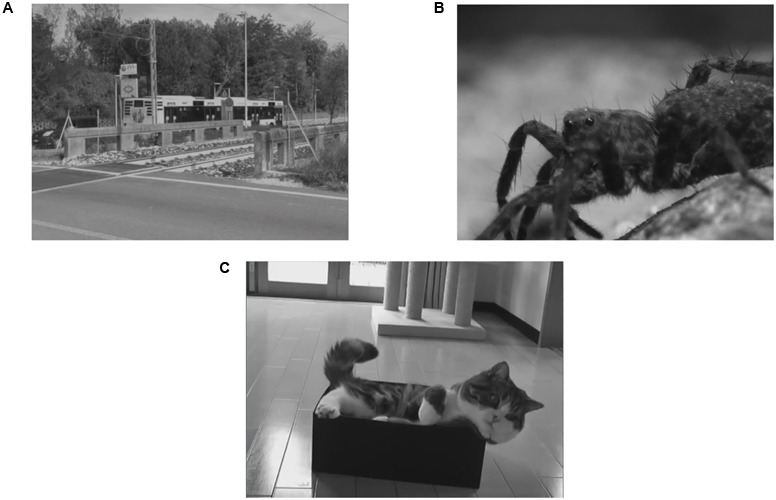
Examples of scenes. **(A)** Neutral condition, **(B)** fearful condition, **(C)** happy condition.

As object shots we used 48 dynamic scenes (gray-scaled and with sound removed), each of 3 s length, representing three emotional conditions: Neutral (*N* = 16), Fear (*N* = 16) and Happiness (*N* = 16). The scenes were previously validated regarding their emotional content. For the happy condition they comprised contents such as puppies, kittens, or newborns. For the fearful condition they included potentially dangerous animals (e.g., spiders, snakes, or a growling dog) or situations (e.g., war scenes). The neutral contexts were mostly provided by city and country views (**Figure 1**) (for details regarding validation procedure and selection criteria, please see Supplementary Materials).

##### Final stimuli

As a final step, we produced the 6-s long film sequences to be used during the experiment by joining the three different shots: the close-up of a target person’s neutral face (glance shot) presented for 1500 ms, followed by a view of the scene or event that the target person was looking at (object shot), presented for a longer duration (3000 ms) in accordance with the Average Shot Length (ASL) in mainstream Hollywood films of between 3 and 4 s (see Salt, 1974; Cutting et al., 2011; Barratt et al., 2016), followed by another close-up of the target person’s neutral face (glance shot) presented for 1500 ms. The final film sequences were presented in Audio Video Interleave (AVI) format and the resolution of the image was 640 pixels × 480 pixels.

For each participant, we created a list of 18 film sequences in total, six per emotional condition (in accordance with the emotion evoked by the object shot) taking into account a few basic rules: each facial identity had to be shown only once; both the gender and the orientation of the faces had to be balanced. Hence, the 18 experimental trials comprised nine trials with female faces (six looking to the left and three looking to the right) and nine trials with male faces (three looking to the left and six looking to the right).

#### Procedure

One day before the experimental session, participants were asked to fill in the following questionnaires via Google Forms: the Toronto Alexithymia Scale (TAS), which measures the ability to identify and describe emotions and feelings, and has three subscales [Difficulty Describing Feelings (DDF), Difficulty Identifying Feelings (DIF) and Externally-Oriented Thinking (EOT)] (Bagby et al., 1994); the Interpersonal Reactivity Index-IRI, which assesses the empathic abilities of each participant, and has four subscales [Perspective Taking (PT), Fantasy (FS), Empathic Concern (EC) and Personal Distress (PD)] (Davis, 1980); and the Behavioral Activation System/Behavioral Inhibition System-BIS/BAS, which measures individual differences in the sensitivity of these systems, and has one BIS-related scale and three BAS-related scales (BAS Drive, BAS Fun Seeking and BAS Reward Responsiveness) (Carver and White, 1994). Once participants arrived at the laboratory and became comfortable, they were also asked to fill in the State-Trait Anxiety Inventory STAI (Spielberger et al., 1970), to assess the level of anxiety of each participant as a permanent trait and/or as contextual. In sum, we asked participants to fill in these questionnaires to exclude the possibility that personality traits or deficits in emotion recognition and empathic abilities could influence the performance in the task.

The experimental procedure included two blocks. In the first experimental block, participants were shown 18 film sequences in random order, and were instructed to rate both the valence and arousal of the target person’s emotion by means of a 9-point scale ranging from -4 (“negative”) to +4 (“positive”) for valence, and from 1 (“calm”) to 9 (“excited”) for arousal (Barratt et al., 2016). Each trial consisted of a black fixation cross on a gray background (500 or 1000 ms), followed by the film-sequence presented for 6 s. At the end of the film sequence, participants were first asked to rate the valence of the target person’s emotion, and then to rate the arousal using the keyboard positioned in front of them and without time limits. A green background was used as inter-trial interval (ITI) with a duration of either 1000 or 1500 ms.

**FIGURE 2:**
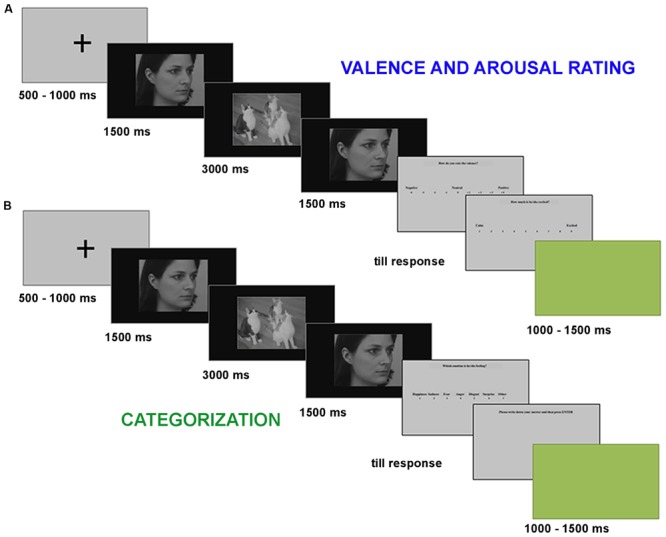
Experimental paradigm. **(A)** valence and arousal rating, **(B)** categorization.

In the second experimental block, participants saw each film sequence one more time (for a total of 18 trials) in a different randomized order and were asked to explicitly categorize the emotion displayed by the target person’s face, choosing among seven categories (happiness, sadness, fear, anger, disgust, surprise, and “other option”). They articulated their choice by using the keyboard positioned in front of them. Again, no time limit was given. When they chose the “other” option, they were asked to write down which was in their opinion the perceived emotion (**Figure 2**).

The experimental session was preceded by a training session that included four trials, showing film sequences edited using scenes excluded at the end of the validation process (two neutral, one happy, and one fearful), and other four facial identities (two female) taken from the KDEF, half of them looking to the left and the other half to the right.

Stimuli delivery and response recording were controlled using E-prime 2.0 software.

At the end of the procedure, the participants were asked to answer five open questions via Google Forms to assess their experience and their familiarity with the stimuli: (1) Have you ever seen some of these videos before? (2) What do you think the experiment was about? (3) Was there anything confusing in the experiment? (4) What was your impression of the different faces?; (5) Do you have any other comments? (6) Have you heard of the Soviet filmmaker Lev Kuleshov and/or the “Kuleshov effect”?

#### Differences from the Original Paradigm of Barratt et al. (2016)

In sum, in contrast to the original paradigm developed by Barratt et al. (2016): (1) we employed new contextual stimuli (and all of them were dynamic scenes); (2) the emotional contexts belonged to only two emotional conditions (happy and fearful; see section “Introduction” for a detailed explanation); (3) we ran two experimental sessions (dimensional vs. categorical evaluation); (4) we added a neutral condition without the “null condition” (no context); (5) facial orientation was counterbalanced across stimuli; (6) we employed different presentation times; (7) we added an ITI; and (8) stimuli had a different dimension.

## Statistical Analysis and Results

In accordance with the previous study by Barratt et al. (2016), we rescaled the valence and arousal scores for each participant so that a value of zero corresponded to the mean rating across all three conditions, respectively. This was done in order to evaluate whether, for each participant, a condition mean was higher (positive value) or lower (negative value) than the overall mean in terms of valence and arousal.

In order to investigate the modulation of rating by context condition, we performed a linear mixed effects analysis. We entered the rating score as a dependent variable, and Measure (2 levels: Arousal and Valence) and Context (3 levels: Neutral, Fearful, and Happy) as independent fixed variables. We entered intercepts for stimuli and subjects, and by-subject slopes for the effect of Context as random effects.

Tukey’s test was used for *post hoc* comparisons among means. Visual inspection of residual plots did not reveal any obvious deviations from homoscedasticity or normality. *P*-values were obtained by likelihood ratio tests of the full model with the effect in question against the model without that effect (Winter, 2013).

Regarding the categorization task, we computed the percentage of answers given by participants to each emotion category for each emotional condition (Happiness, Sadness, Fear, Anger, Disgust, Surprise, and Other emotion). For all analyses, we used R (R Core Team, 2012) and lmerTest (Kuznetsova et al., 2015).

### Results

The model [χ^2^(2) = 143.68, *p* < 0.0001] explained 10% of the variance in score, not taking into account the random effects (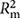 = 0.10; 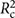 = 0.21). Results for random effects showed that the variability explained by “stimulus” was <0.0001 and the variability explained by “subject” was <0.25.

We observed a main effect of Measure with Valence scores being significantly different from the intercept and on average being higher than Arousal scores (β = 0.78, *SE* = 0.2, *t* = 3.9, *p* < 0.0001). The model revealed a main effect of Context (across both measures) with neutral faces in the fearful context on average being rated 1.29 point higher than neutral faces in the neutral context (β = 1.29, *SE* = 0.2, *t* = 5.9, *p* < 0.0001), while neutral faces in the happy context did not differ from neutral faces in the neutral context (β = 0.33, *SE* = 0.2, *t* = 1.4, *p* = 0.17). The model also revealed a significant Measure^∗^Context interaction effect (β = -2.54, *SE* = 0.3, *t* = -8.9, *p* < 0.0001). *Post hoc* tests showed that all comparisons between measures within each context were significant (Neutral = Arousal: *M* = -0.54, *SE* = 0.17; Valence: *M* = 0.24, *SE* = 0.17; Fear = Arousal: *M* = 0.75, *SE* = 0.19; Valence: *M* = -1.01, *SE* = 0.19; Happiness = Arousal: *M* = -0.21, *SE* = 0.2; Valence: *M* = 0.76, *SE* = 0.2; all *P*s < 0.0001). This is just an elaboration to make the findings of the interaction of Measure^∗^Condition more clear. More important to our hypothesis are the next two findings. Considering Arousal scores, neutral faces in the fearful context were rated 1.29 point higher than neutral faces in the neutral context (*p* < 0.0001), and 0.96 point higher than neutral faces in the happy context (*p* < 0.0001). There was not a significant difference between Neutral and Happiness.

Considering Valence scores, neutral faces in the fearful context were rated -1.25 point lower than neutral faces in the neutral context (*p* < 0.0001), and -1.77 point lower than neutral faces in the happy context (*p* < 0.0001).

**FIGURE 3:**
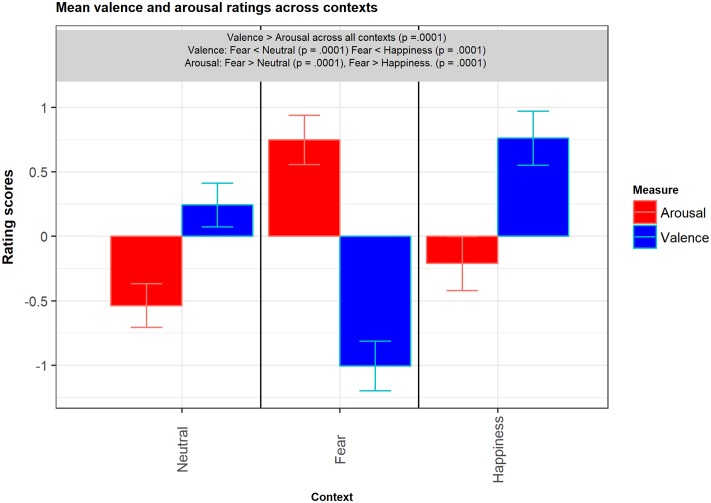
Bar plots of mean valence and arousal ratings across contexts. Error bars represent SE. Significant differences indicated in box above bars.

*Post hoc* tests did not reveal a significant difference between neutral faces in the neutral context and neutral faces in the happy context for both the Valence and Arousal measures (**Figure 3**).

#### Categorization

If the emotional contexts had no effect on the emotional attribution of the target person’s emotional state (the null hypothesis), then each of the seven categories should have been selected with an equal degree of probability (Barratt et al., 2016); that is, a relative frequency approaching 14.3%.

For the fear context, participants tended to choose negative emotions more frequently than the other options, with fear being the most selected answer (Fear = 24.4%, Sadness = 22.6%, Other = 16.1%, Disgust = 14.8%, Surprise = 9.5%, Anger = 7.7%, and Happiness = 4.8%) (**Table 1**).

**Table 1:** Percentages expressing the relative frequency of the selection of the seven categories for each emotional context (Fear, Happy, and Neutral).

	Happiness	Sadness	Fear	Anger	Disgust	Surprise	Other
Fear context	4.8%	22.6%	24.4%	7.7%	14.8%	9.5%	16.1%
Happy context	35.7%	11.3%	5.9%	6.5%	2.9%	20.2%	17.3%
Neutral context	20.2%	25%	11.3%	4.2%	1.2%	14.3%	23.8%

For the happiness context condition, participants tended to choose positive emotions more frequently than the other options (Happiness = 35.7%, Surprise = 20%, Other = 17.2%, Sadness = 11%, Anger = 6.5%, Fear = 6%, and Disgust = 3%).

For the neutral condition, participants tended to choose the “other” option more frequently than the other options (Other = 24%) (**Table 1**).

### Assessment

The mean values obtained on each questionnaire did not reveal the presence of participants with personality traits or deficits in emotion recognition and/or in empathic abilities:

TAS-20: the mean *DDF* subscale score ± *SD* was 13.8 ± 5; the mean *DIF* subscale score ± *SD* was 16.1 ± 7.4; the mean *EOT* subscale score ± *SD* was 14.6 ± 4.4; the mean *total* score ± *SD* was 44.4 ± 14.6;

IRI: the mean *EC* score ± *SD* was 21.2 ± 3.1; the mean *PD* score ± *SD* was 10.8 ± 5.6; the mean *PT* score ± *SD* was 19.4 ± 4.5; the mean *FS* score ± *SD* was 17 ± 5.1.

BIS/BAS: the mean *BIS* score ± *SD* was 25.4 ± 3.2; the mean *BAS Drive* score ± *SD* was 13 ± 3.4; the mean *BAS Fun Seeking* score ± *SD* was 12 ± 3.8; the mean *BAS Reward Responsiveness* score ± *SD* was 21 ± 2.5;

STAI: the mean *STAI X2 Trait* score ± *SD* was 41.8 ± 9.2; the mean *STAI X1 Pre* score ± *SD* was 33.8 ± 6.8; the mean *STAI X1 Post* score ± *SD* was 17.4 ± 5.4.

## Discussion

The aim of the present study was to investigate the influence of contextual cues on our evaluation of facial expressions of emotion. In order to do this, we connected the field of research on emotion perception with the field of research on the perception of films. Specifically, we aimed at replicating the Kuleshov effect (e.g., Pudovkin, 1970; Kuleshov, 1974) by means of an improved experimental design based on Barratt et al., (2016), introducing some modifications which, in our opinion, added value to the experimental design (see section “Materials and Methods” for details).

In order to study the context-sensitivity of emotions under more ecologically valid conditions, we used dynamic scenes as contextual stimuli. Participants were shown 18 film sequences of neutral faces across three emotional contexts conditions (Neutral, Happiness, and Fear). The task was to rate the emotion displayed by a neutral target person’s face in terms of valence, arousal, and category. Hence, we adopted both a dimensional and a categorical approach to emotion (e.g., Ekman and Friesen, 1971; e.g., Russell, 1980) in order to have as much information as possible about the participants’ experience.

Our results confirmed the presence of a significant effect in terms of both valence and arousal for the Fear context only. More specifically, participants rated neutral faces in fearful contexts as significantly more negative and more arousing than neutral faces in both neutral or happy contexts. Moreover, participants tended to categorize the target person’s facial expressions choosing the emotion categories appropriate with the preceding context (positive emotions for the Happiness condition vs. negative emotions for the Fear condition). Hence, while from a dimensional point of view our results suggest the presence of a significant effect when neutral faces were paired with fearful contexts, from a categorical point of view our participants tended to choose the emotion categories congruent with the preceding context also when neutral faces were paired with happy contexts.

On the basis of the affective prediction hypothesis (Barrett and Bar, 2009; Barrett et al., 2011) the Kuleshov effect could be explained by a mechanism which detects the visual sensations of the emotional context and interprets them by means of the corresponding affective representations, generating a prediction to signal neutral faces as emotional faces. In our view, however, a more suitable explanation for the Kuleshov effect is that the context triggers the arousal and the emotional reaction in the observer who then attributes an emotional value to a neutral face.

More specifically, our results differ from Barratt et al., (2016) findings in the following terms: while Barratt et al. demonstrated the presence of the effect only when faces were paired with contexts of desire or sadness, our results also showed a significant effect when faces were paired with fearful contexts, but not if they were paired with happy contexts. As stated before, we employed only two emotional contexts (Happy and Fearful) in order to keep the design as simple as possible and to highlight the differences between opposite emotional conditions (see section “Introduction”). More specifically, we adopted fear as a negative emotion because, from an evolutionary point of view, it is capable of directing our attention to potentially dangerous stimuli (such as the scenarios depicted in our fearful contexts). In this regard, an interesting explanation is provided by the *motivated attention theory* (Lang et al., 1997; Bradley et al., 2003) also stating that cues that signal danger activate one of the two major motivation circuits (defensive vs. appetitive motivational systems; e.g., Bradley et al., 2001a,b; Lang and Bradley, 2010) pushing amplified orienting and attention responses (Bradley et al., 2012). Moreover, since the activation of these motivational circuits can be elicited also by pictures (e.g., Bradley et al., 2012), the “defensive” response is amplified when phobic or fearful individuals view fear-related pictures (Globisch et al., 1999; Sabatinelli et al., 2001; Bradley et al., 2012). Additionally, it has been demonstrated that this aversive response, defined by modulations in self-report, physiological, and behavioral systems (e.g., Lang et al., 1993; Sabatinelli et al., 2001), could also persist after slide offset (e.g., Globisch et al., 1999). We suggest that the same mechanisms are elicited when using fear-related videos, thus explaining our results. For these reasons, future studies aiming to assess this effect using fearful and phobic contexts should include an evaluation of phobic traits by means of dedicated questionnaires (e.g., Snake and Spider questionnaires, SNAQ and SPQ, respectively; Klorman et al., 1974).

The absence of a significant modulation of valence and arousal ratings when neutral faces were paired with happy contexts could be ascribed to the kind of positive scenarios we proposed to our participants. Indeed, among stimuli rated as pleasant, erotic materials elicit the strongest affective reactions (Bradley et al., 2001a,b; Sabatinelli et al., 2001, p. 719). As a matter of note, Barratt et al. (2016) demonstrated a significant effect exactly with desire contexts. In our opinion, altogether these results seem to suggest that this kind of contextual effect emerges more clearly when employing strong arousing emotional contexts as stimuli. Future studies should further clarify this aspect.

Taken together, our results again highlight the context-sensitivity of emotions and the importance of studying them under ecologically valid conditions.

### Future Directions

A goal for future studies will be to investigate this effect in different modalities, creating auditory emotional contexts to distinguish the capability of visual and auditory modalities to influence the comprehension of facial expressions. As far as we know, there has been only one previous study dedicated to investigating *the role of sound in the evaluation of facial expressions in films* using Kuleshov-type experimental sequences (Baranowski and Hecht, 2017, p. 624). They asked participants to rate the emotional state of the actor on the six basic emotions, thus adopting a categorical approach only. Moreover, they employed an experimental design suitable for investigating the multisensory integration of music and facial expressions but for this reason different from the original Kuleshov sequences. Thus, despite their encouraging results, future studies should further assess the role of the auditory modality on the comprehension of facial expressions.

Moreover, since little has been done to explore such contextual modulations on emotion processing at the physiological level, in order to further investigate questions about the interaction between contextual cues and the comprehension of facial expressions, it would be important to use time sensitive measures, such as electroencephalography (EEG) (Wieser and Brosch, 2012). We think that our advanced and more ecological design will be of great help in developing new studies to better understand emotion processing in humans.

## Author Contributions

MC, KH, and DB designed the experiment. MC, KH, and FS performed data acquisition and analyses. MC, KH, DB, FS, MU, and VG interpreted the results. MC wrote the paper. All authors have contributed to, seen and approved the manuscript.

## Conflict of Interest Statement

The authors declare that the research was conducted in the absence of any commercial or financial relationships that could be construed as a potential conflict of interest.

## References

[B1] Aviezer H. Hassin R. R. Ryan J. Grady C. Susskind J. Anderson A. (2008). Angry, disgusted, or afraid?: studies on the malleability of emotion perception. *Psychol. Sci.* 19 724–732. 10.1111/j.1467-9280.2008.02148.x 18727789

[B2] Bagby R. M. Parker J. D. Taylor G. J. (1994). The twenty-item Toronto Alexithymia Scale-I. Item selection and cross-validation of the factor structure. *J. Psychosom. Res.* 38 23–32. 10.1016/0022-3999(94)90005-1 8126686

[B3] Baranowski A. M. Hecht H. (2017). The auditory Kuleshov effect: multisensory integration in movie editing. *Perception* 46 624–631. 10.1177/0301006616682754 27923940

[B4] Barratt D. Rédei A. C. Innes-Ker Å van de Weijer J. (2016). Does the Kuleshov effect really exist? Revisiting a classic film experiment on facial expressions and emotional contexts. *Perception* 45 847–874. 10.1177/0301006616638595 27056181

[B5] Barrett L. F. Bar M. (2009). See it with feeling: affective predictions during object perception. *Phil. Trans. R. Soc. B* 364 1325–1334. 10.1098/rstb.2008.0312 19528014 PMC2666711

[B6] Barrett L. F. Lindquist K. A. Gendron M. (2007). Language as context for the perception of emotion. *Trends Cogn. Sci.* 11 327–332. 10.1016/j.tics.2007.06.003 17625952 PMC2225544

[B7] Barrett L. F. Mesquita B. Gendron M. (2011). Context in emotion perception. *Curr. Dir. Psychol. Sci.* 20 286–290. 10.1177/0963721411422522

[B8] Boll S. Gamer M. Kalisch R. Buchel C. (2011). Processing of facial expressions and their significance for the observer in suregions of the human amygdala. *Neuroimage* 56 299–306. 10.1016/j.neuroimage.2011.02.021 21320610

[B9] Bradley M. M. Codispoti M. Cuthbert B. N. Lang P. J. (2001a). Emotion and motivation I: defensive and appetitive reactions in picture processing. *Emotion* 3 276–298. 12934687

[B10] Bradley M. M. Codispoti M. Sabatinelli D. Lang P. J. (2001b). Emotion and motivation II: sex differences in picture processing. *Emotion* 1 300–319. 12934688

[B11] Bradley M. M. Keil A. Lang P. J. (2012). Orienting and emotional perception: facilitation, attenuation, and interference. *Front. Psychol.* 3:493 10.3389/fpsyg.2012.00493 PMC3499912 23181039

[B12] Bradley M. M. Sabatinelli D. Lang P. J. Fitzsimmons J. R. King W. Desai P. (2003). Activation of the visual cortex in motivated attention. *Behav. Neurosci.* 117 369–380. 10.1037/0735-7044.117.2.369 12708533

[B13] Branigan E. (1984). *Point of View in the Cinema.* New York, NY: Mouton 10.1515/9783110817591

[B14] Brewster B. (1982). A scene at the ‘movies’. *Screen* 23 4–15. 10.1093/screen/23.2.4

[B15] Calder A. J. Ewbank M. Passamonti L. (2011). Personality influences the neural responses to viewing facial expressions of emotion. *Philos. Trans. R. Soc. Lond. B Biol. Sci.* 366 1684–1701. 10.1098/rstb.2010.0362 21536554 PMC3130379

[B16] Carroll J. M. Russell J. A. (1996). Do facial expressions signal specific emotions? Judging emotion from the face in context. *J. Pers. Soc. Psychol.* 70 205–218. 10.1037/0022-3514.70.2.205 8636880

[B17] Carroll N. (ed.) (1993). “Toward a theory of point-of-view editing: communication, emotion, and the movies,” in *Theorizing the Moving Image* (Cambridge: Cambridge University Press), 125–138. 10.2307/1773144

[B18] Carver C. S. White T. L. (1994). Behavioral inhibition, behavioral activation, and affective responses to impending reward and punishment: the BIS/BAS Scales. *J. Pers. Soc. Psychol.* 67 319–333. 10.1037/0022-3514.67.2.319

[B19] Cutting J. E. Brunick K. L. De Long J. E. Iricinschi C. Candan A. (2011). Quicker, faster, darker: changes in hollywood film over 75 years. *Iperception* 2 569–576. 10.1068/i0441aap 23145246 PMC3485803

[B20] Davis M. H. (1980). A multidimensional approach to individual differences in empathy. *JSAS Cat. Sel. Doc. Psychol.* 10 85.

[B21] de Gelder B. Meeren H. K. M. Righart R. van den Stock J. van de Riet W. A. Tamietto M. (2006). Beyond the face: exploring rapid influences of context on face processing. *Prog. Brain Res.* 155 37–48. 10.1016/S0079-6123(06)55003-4 17027378

[B22] Ekman P. (1992). An argument for basic emotions. *Cogn. Emot.* 6 169–200. 10.1080/02699939208411068

[B23] Ekman P. (1993). Facial expressions and emotion. *Am. Psychol.* 48 384–392. 10.1037/0003-066X.48.4.384 8512154

[B24] Ekman P. Friesen W. (1971). Constants across cultures in the face and emotion. *J. Pers. Soc. Psychol.* 17 124–129. 10.1037/h0030377 5542557

[B25] Fridlund A. J. (1994). *Human Facial Expression: An Evolutionary View.* San Diego, CA: Academic Press.

[B26] Globisch J. Hamm A. O. Esteves F. Öhman A. (1999). Fear appears fast: temporal course of startle reflex potentiation in animal fearful subjects. *Psychophysiology* 36 66–75. 10.1017/S0048577299970634 10098381

[B27] Hajcak G. Weinberg A. MacNamara A. Foti D. (2011). “ERPs and the study of emotion,” in *The Oxford Handbook of ERP Components*, eds Luck S. J. Kappenman E. S. (New York, NY: Oxford University Press).

[B28] Izard C. E. (1994). Innate and universal facial expressions: evidence from developmental and cross-cultural research. *Psychol. Bull.* 115 288–299. 10.1037/0033-2909.115.2.288 8165273

[B29] Kim H. Somerville L. H. Johnstone T. Polis S. Alexander A. L. Shin L. M. (2004). Contextual modulation of amygdala responsivity to surprised faces. *J. Cogn. Neurosci.* 16 1730–1745. 10.1162/0898929042947865 15701225

[B30] Klorman R. Weerts T. C. Hastings J. E. Melamed B. G. Lang P. J. (1974). Psychometric description of some specific-fear questionnaires. *Behav. Ther.* 5 401–409. 10.1016/S0005-7894(74)80008-0

[B31] Kuleshov L. (1974). *Kuleshov on Film*, ed. Levaco R. (Berkeley, CA: University of California Press).

[B32] Kuznetsova A. Brockhoff P. B. Christensen R. H. B. (2015). *Package “lmerTest”. R Package Version.* Available at: http://www.rdocumentation.org/packages/lmerTest

[B33] Lang P. Bradley M. M. (2007). “The International Affective Picture System (IAPS) in the study of emotion and attention,” in *Handbook of Emotion Elicitation and Assessment*, eds Coan J. A. Allen J. J. B. (Oxford: Oxford University Press), 29.

[B34] Lang P. J. Bradley M. M. (2010). Emotion and the motivational brain. *Biol. Psychol.* 84 437–450. 10.1016/j.biopsycho.2009.10.007 19879918 PMC3612949

[B35] Lang P. J. Bradley M. M. Cuthbert B. N. (1997). “Motivated attention: affect, activation, and action,” in *Attention and Orienting*, eds Lang P. J. Simons R. F. Balabon M. (Mahwah, NJ: Erlbaum), 97–135.

[B36] Lang P. J. Greenwald M. K. Bradley M. M. Hamm A. O. (1993). Looking at pictures: affective, facial, visceral, and behavioral reactions. *Psychophysiology* 30 261–273. 10.1111/j.1469-8986.1993.tb03352.x 8497555

[B37] Lundqvist D. Flykt A. Öhman A. (1998). *The Karolinska Directed Emotional Faces – KDEF (CD-ROM).* Stockholm: Karolinska Institutet.

[B38] Meeren H. K. M. van Heijnsbergen C. C. R. J. de Gelder B. (2005). Rapid perceptual integration of facial expression and emotional body language. *Proc. Natl. Acad. U.S.A.* 102 16518–16523. 10.1073/pnas.0507650102 PMC1283446 16260734

[B39] Mobbs D. Weiskopf N. Lau H. C. Featherston E. Dolan R. J. Frith C. D. (2006). The Kuleshov effect: the influence of contextual framing on emotional attributions. *Soc. Cogn. Affect. Neurosci.* 1 95–106. 10.1093/scan/nsl014 17339967 PMC1810228

[B40] Persson P. (2003). *Understanding Cinema: A Psychological Theory of Moving Imagery.* Cambridge: Cambridge University Press 10.1017/CBO9780511497735

[B41] Prince S. Hensley W. E. (1992). The Kuleshov effect: recreating the classic experiment. *Cinema J.* 31 59–75. 10.2307/1225144

[B42] Pudovkin V. I. (1970). *Film Technique and Film Acting*, ed. Montagu I. (New York, NY: Grove Press, Inc.).

[B43] R Core Team (2012). *R: A Language and Environment for Statistical Computing.* Vienna: R Foundation for Statistical Computing.

[B44] Russell J. A. (1980). The circumplex model of affect. *J. Pers. Soc. Psychol.* 39 1161–1178. 10.1037/h0077714

[B45] Russell J. A. (1997). “Reading emotions from and into faces: resurrecting a dimensional contextual perspective,” in *The Psychology of Facial Expressions*, eds Russell J. A. Fernandez J. M.-Dols (New York, NY: Cambridge University Press), 295–320.

[B46] Sabatinelli D. Bradley M. M. Lang P. J. (2001). Affective startle modulation in anticipation and perception. *Psychophysiology* 38 719–722. 10.1111/1469-8986.3840719 11446586

[B47] Salt B. (1974). Statistical style analysis of motion pictures. *Film Q.* 28 13–22. 10.2307/1211438

[B48] Schwarz K. A. Wieser M. J. Gerdes A. B. M. Mühlberger A. Pauli P. (2013). Why are you looking like that? How the context influences evaluation and processing of human faces. *Soc. Cogn. Affect. Neurosci.* 8 438–445. 10.1093/scan/nss013 22287265 PMC3624952

[B49] Spielberger C. D. Gorsuch R. L. Lushene R. E. (1970). *Manual for the State-Trait Anxiety Inventory.* Palo Alto, CA: Consulting Psychologists Press.

[B50] Tomkins S. S. (1962–1963). *Affect, Imagery, Consciousness*, Vol. 1 and 2 New York, NY: Springer.

[B51] Wieser M. J. Brosch T. (2012). Faces in context: a review and systematization of contextual influences on affective face processing. *Front. Psychol.* 3:471 10.3389/fpsyg.2012.00471 PMC3487423 23130011

[B52] Winter B. (2013). Linear models and linear mixed effects models in R with linguistic applications. *arXiv* arxiv:1308.5499.

