# Supplementary material for: How Context Influences Our Perception of Emotional Faces: A Behavioral Study on the Kuleshov Effect
Source: Front Psychol. 2017 Oct 4;8:1684. doi: 10.3389/fpsyg.2017.01684 (PMC5632723; doi:10.3389/fpsyg.2017.01684)
Supplement: Supplementary file 1 [file Data_Sheet_1.docx]

Supplementary Material

**How context influences our perception of emotional faces: a behavioral study on the Kuleshov effect.**

**Marta Calbi^*^, Katrin Heimann, Daniel Barratt, Francesca Siri, Maria Alessandra Umiltà, Vittorio Gallese.**

*** Correspondence:** Corresponding Author: [calbimarta@gmail.com](mailto:calbimarta@gmail.com)

# Supplementary Data

**Validation procedure**

**Participants**

Twenty-two healthy adult volunteers took part in the validation process (12 female and 10 male; age range: 22-42 years; mean age: 30.4; SD = 5.04).

**Stimuli**

The object shots were based on 97 videos obtained through a YouTube and Vimeo search. They were split into three emotional conditions: Neutral (N = 32), Happiness (N = 33), and Fear (N = 33). We mainly focused on high-resolution videos involving good quality camera movements made from either left to right or right to left on the horizontal plane. By means of “Final cut Pro X 10.2.3” (2001/2006 Apple inc.), we selected 3 seconds considered as the most representative of the emotional condition they belonged to. Each scene was then converted to greyscale, and 640 x 480 pixels (standard 4:3 aspect ratio), and the sound was removed. In order to test the validity of the object shots (i.e., to ensure that they were easily comprehensible in terms of their intended emotions), the object shots were randomly presented to participants by means of an online survey on Survey Monkey (Survey Monkey Inc., Palo Alto, California, USA; www.surveymonkey.com), and the participants were asked to rate the emotion evoked by each of the 3-second scenes in terms of valence, arousal and category. In order to assess the emotional reaction in terms of valence and arousal, we used a picture-oriented 7-point scale, the Self-Assessment Manikin Scale (SAM), an affective rating system devised by Lang (1994). The non-verbal scale SAM used for the valence rating ranged from a frowning, unhappy figure to a smiling and happy figure, while for the arousal dimension ranged from a calm and relaxed figure to an excited figure. Hence, we asked participants to click on the SAM scale according to the mood and sensations elicited by each scene. To assess the emotional categorization, participants were asked to choose from seven different categories the one they considered most representative of the emotion evoked by each scene: neutral, sadness, happiness, surprise, anger, fear, or disgust.

**Selection criteria**

To select the scenes to be used during the behavioral experiment, we adopted a criteria based on the numerical values of the 7-point valence SAM scale. As fearful stimuli, we selected the ones with a valence score below 2.5 (N=16), as happy stimuli the ones with a valence score above 5.5 (N=16), and as neutral stimuli the ones with a valence score between 3.5 and 4.5 (N=16) (see Supplementary Table 1). Since we also asked the participants to explicitly categorize the emotion evoked by each video, we attributed numerical values to participants’ answers: -1 for all negative emotions (sadness, anger, fear, and disgust), 1 for happiness, 0.5 to surprise, and 0 to neutral. For each stimulus, we then computed the mean and we rejected negative stimuli with an average value above -0.5, happy stimuli with an average value below 0.5, and neutral stimuli with an average value either above 0.5 or below -0.5. All of these stimuli were excluded because they were outside the border of the valence cut-off (see above). At the end of this procedure, we were able to select 16 object shots for each emotional condition from the initial total of 97 scenes.

**Statistical analysis and results**

In order to validate our selected object shots (scenes or events), we tested the influence of condition (Happiness, Fear, Neutral) on the dependent variables (arousal and valence score, respectively and separately) by using R (R Core Team, 2012) and lmerTest (Kuznetsova, Brockhoff and Christensen, 2015) to perform a linear mixed model analysis. We entered intercepts for subjects and stimuli as random effects. Tukey’s test was used for post-hoc comparisons between means. Visual inspection of residual plots did not reveal any obvious deviations from homoscedasticity or normality. P-values were obtained by likelihood ratio tests of the full model with the effect in question against the model without that effect (Winter, 2013). We expected that participants were more likely to report lower, higher, and intermediate valence scores for the Happiness, Fear and Neutral scenes respectively. In addition, we predicted that participants were more likely to experience higher levels of arousal in happy and fear condition in contrast to neutral condition.

**Results**

Arousal

The model (χ2(2)=81.633, p <.0001) explained 25% of the variance in score, not taking into account the random effects (R2m=.25; R2c=.50). We observed that 32% of the variability in the dependent measure (arousal score) was due to stimuli, while 80% was due to subjects. The analysis revealed that the predicted main effect of condition was significant (p < .0001). Specifically, scenes belonging to the Fear condition were rated 2.15 points higher than Neutral scenes (β=2.15, SE=0.15, t=14.6, p < .0001). Scenes belonging to the Happiness condition were rated 0.8 points higher than scenes from the Neutral category (β=0.8, SE=0.15, t=5.24, p = <. 001). All conditions were significantly different from each other (p < .0001).

Valence

The model (χ2(2)=186, p < .0001) explained 73% of the variance in score, not taking into account the random effects (R2m=.73; R2c=.76). We observed that 12% of the variability in the dependent measure (valence score) was due to stimuli, while 31% was due to subjects. The analysis revealed that the predicted main effect of condition was significant (p < .0001). Specifically, scenes belonging to the Happiness condition elicited a positive rating (M = 5.88, SE = 0.08), while Fear scenes evoked a less positive rating (M = 2 .11; SE = 0.08) and Neutral scenes had an intermediate rating (M = 4.02; SE = 0.08). As shown by contrasts, the rating of the scenes belonging to Fear condition were significantly different from the Neutral condition as they were rated -1.90 points lower than the Neutral category (β=-1.90, SE=0.08, t=-24.31, p < .0001). Moreover, the Happiness scenes were rated 1.85 points higher than scenes from the Neutral condition (β=1.85, SE=0.08, t=23.6, p < .0001). All conditions were significantly different from each other (p < .0001).

Considered altogether, these results demonstrate that the selected stimuli belong to and evoke the three emotions of interest (Neutral, Happiness, and Fear).

**Bibliography**

Bradley, M. M., & Lang, P. J. (1994). Measuring emotion: The self-assessment manikin and the semantic differential. *Journal of Behavioral Therapy and Experimental Psychiatry*, 25, 49-59.

Kuznetsova, A., Brockhoff, P.B., Christensen, R.H.B. (2015). Package “lmerTest”. R package version. Available at: http://www.rdocumentation.org/packages/lmerTest

Winter, B. (2013). Linear models and linear mixed effects models in R with linguistic applications.

arXiv:1308.5499. [http://arxiv.org/pdf/1308.5499.pdf]

**2 Supplementary Table**

| **Neutral Stimuli** | **Valence Score (SD)** | **Fear**  **Stimuli** | **Valence Score**  **(SD)** | **Happy**  **Stimuli** | **Valence Score (SD)** |
| --- | --- | --- | --- | --- | --- |
| **n68** | 3.82 (1.01) | **f40** | 1.32 (0.57) | **h36** | 6.09 (1.02) |
| **n45** | 3.86 (0.56) | **f39** | 1.55 (0.67) | **h1** | 6.05 (1.09) |
| **n30** | 3.86 (0.83) | **f37** | 1.82 (0.91) | **h4** | 6.05 (0.84) |
| **n55** | 3.91 (0.43) | **f32** | 1.91 (0.75) | **h8** | 6.00 (0.87) |
| **n43** | 3.91 (0.81) | **f27** | 1.91 (1.11) | **h7** | 6.00 (0.76) |
| **n56** | 3.95 (0.58) | **f31** | 1.95 (0.84) | **h22** | 5.95 (0.90) |
| **n48** | 4.00 (0.93) | **f30** | 2.05 (1.05) | **h32** | 5.95 (1.00) |
| **n57** | 4.05 (0.49) | **f29** | 2.14 (0.99) | **h5** | 5.86 (1.13) |
| **n44** | 4.05 (0.79) | **f21** | 2.27 (1.03) | **h33** | 5.86 (1.08) |
| **n51** | 4.09 (0.68) | **f1** | 2.36 (1.22) | **h40** | 5.82 (1.14) |
| **n22** | 4.09 (0.87) | **f36** | 2.36 (1.14) | **h19** | 5.77 (1.02) |
| **n50** | 4.09 (0.75) | **f28** | 2.36 (1.76) | **h35** | 5.77 (0.87) |
| **n39** | 4.09 (0.43) | **f6** | 2.41 (0.73) | **h2** | 5.73 (1.08) |
| **n54** | 4.14 (0.64) | **f10** | 2.41 (1.05) | **h23** | 5.73 (1.16) |
| **n61** | 4.18 (0.80) | **f22** | 2.45 (1.02) | **h21** | 5.73 (0.98) |
| **n60** | 4.23 (0.87) | **f16** | 2.50 (1.14) | **h31** | 5.64 (1.00) |

**Supplementary Table 1:** valence score and SD of the selected stimuli.
